# Effects of Wushu Programs on Lower-Limb Explosive Power in Preschool Children Aged 5–6 Years: A Cluster-Randomized Controlled Trial

**DOI:** 10.3390/jfmk11020222

**Published:** 2026-05-31

**Authors:** Beibei Luo, Ruoxi Fan, Rui Li, Rongda Wang, Xiaomiao Zheng, Rui Huang, Shuxin Zhang, Yiwei Sun, Zhibei Zhou, Yunya Zhang

**Affiliations:** 1 School of Exercise and Health, Shanghai University of Sport, Shanghai 200438, China; 2 School of Athletic Performance, Shanghai University of Sport, Shanghai 200438, China; 3 School of Wushu, Shanghai University of Sport, Shanghai 200438, China

**Keywords:** children, exercise intervention, lower limb strength, standing long jump, Chinese martial arts

## Abstract

**Background:** Wushu, a traditional Chinese exercise, has been demonstrated to be effective in promoting lower-limb strength in children. However, studies comparing the effects of different intervention durations on preschool children remain limited. **Objectives:** The present study examined the short- and long-term effects of Wushu exercise programs on lower-limb explosive power in preschool children aged 5–6 years. **Methods:** This study was conducted across two experiments, with separate cohorts of children. The children were randomly assigned to either an intervention (INT) or a control (CON) group based on their Kindergarten classes. In Experiment 1, the INT-1 group (n = 55) completed a 4-week ‘Twelve Zodiac’ Wushu exercise program, which comprised three 30-minute sessions per week, while the CON-1 group (n = 49) participated in construction and carrying-based unstructured free play, which was designed to provide a comparable amount of moderate-to-vigorous physical activity. In Experiment 2, the INT-2 group (n = 57) undertook a 10-week Wushu program, and the CON-2 group (n = 38) engaged in similar activities as CON-1 for a 10-week period. The standing long jump (SLJ) was the primary outcome measure in both experiments. Secondary outcomes included the double-leg continuous jump, 15 m zigzag run, grip strength, sit-and-reach, and anthropometric measurements. In Experiment 2, countermovement jump (CMJ) and squat jump (SJ) heights were also measured using a force plate as additional secondary outcomes. A linear mixed-effects model (LMM) was used to analyze the data. **Results:** At baseline, no significant outcome measures were observed between CON-1 and INT-1, nor between CON-2 and INT-2. In Experiment 1, SLJ exhibited a significant enhancement in INT-1 in comparison to CON-1 (*p* = 0.007). The INT-2 in Experiment 2 showed significant improvements compared with CON-2 in the SLJ (*p =* 0.048), double-leg continuous jump (*p =* 0.005), and 15 m zigzag run (*p =* 0.043). A strong correlation was observed between SLJ and 15 m zigzag run time (r = −0.53, *p* < 0.001), and between double-leg continuous jump time and 15 m zigzag run time (r = 0.56, *p* < 0.001). **Conclusions:** The findings of this study indicate that 4-week and 10-week Wushu exercise programs enhance explosive power in the lower limbs of children aged 5–6 years. The 10-week Wushu program improves lower limb coordination and jumping agility. These task-specific adaptations support the value of Wushu interventions for fostering comprehensive lower-limb motor competence in preschoolers.

## 1. Introduction

Wushu, often referred to as Chinese martial arts or Kung Fu, is a broad discipline encompassing a wide range of practices, among which Taolu constitutes a central component. The International Wushu Federation (IWUF) defines Taolu as a continuously connected set of predetermined techniques, incorporating hand techniques, leg techniques, jumps, sweeps, stances, footwork, and balances [1]. It is evident that these elements inherently demand repeated lower-limb loading and rapid force generation. Other Wushu modalities, such as Tai Chi, emphasize a range of elements including controlled movements, weight shifting, and repetitive lower-limb loading. A 24-week Tai Chi program has been shown to improve limits of stability and directional control in patients with Parkinson’s disease to a greater extent than resistance training or stretching [2]. In preschool children, Chinese martial arts training has been found to enhance motor skills [3]. The findings indicate that Wushu training possesses an inherent capacity to enhance both the explosive and the stability functions of the lower limbs through coordinated neuromuscular control.

It is essential to note that lower limb muscular fitness is a critical component of motor development in the preschool years. The acquisition of postural control, dynamic balance, and fundamental locomotor skills, such as running and jumping, is strongly associated with perceived physical competence in school-aged children [4]. The standing long jump (SLJ) has been defined as a field test of lower limb explosive power. Its performance has been biomechanically linked to vertical jumps, notably the squat jump (SJ) and countermovement jump (CMJ) [5,6]. Biomechanical analyses have shown that the SLJ can be divided into a preparatory countermovement phase, like the CMJ, and a concentric propulsion phase that resembles the SJ [7,8]. Force plate analysis enables precise measurement of the height of SJ and CMJ, and the eccentric utilization ratio (EUR). These key indicators of neuromuscular control and stretch-shortening cycle (SSC) efficiency [9]. The utilization of these metrics is further substantiated by age-related reference data for CMJ, SJ, and EUR from childhood to adulthood [10]. Consequently, the integration of the SLJ with force-plate assessments of the CMJ, SJ, and EUR yields a more comprehensive picture of lower-limb explosive power in young children [11].

A growing body of evidence documents the benefits of martial arts interventions for physical fitness and motor competence in pediatric populations. Kung Fu training has been reported to improve balance in young adolescents [12]. Recent systematic reviews further confirm that martial arts interventions consistently improve motor competence, muscle strength, balance, and coordination in children and adolescents, including those with developmental coordination disorder [13,14,15]. Beyond martial arts, structured exercise programs of other modalities, such as 12-week integrative neuromuscular training, coordination-based training, and rhythmic physical activities, have been shown to enhance muscular fitness, motor competence, and gross motor development in preschool children [16,17,18], supporting the view that early childhood is a sensitive window for well-designed physical interventions.

Despite the evident benefits of martial arts, many studies have concentrated on overall outcomes such as general fitness or balance, while few have explored whether martial arts training enhances lower-limb explosive power. Furthermore, the use of force-plate measures to quantify jump height and SSC efficiency in preschoolers has been limited. Therefore, the present study examined the effects of a Wushu program on lower-limb explosive power in preschool children through two separate cluster-randomized trials. One implemented a 4-week intervention, and the other a 10-week intervention. The standing long jump was integrated as the primary outcome. The secondary outcomes encompassed lower limb agility, coordination, and force plate-derived SJ height, CMJ height, and EUR. This research provided experimental evidence on the potential of Wushu training to support lower-limb motor development in early childhood.

## 2. Materials and Methods

### 2.1. Study Design

This study was conducted across two experiments, with separate cohorts of children, between 2024 and 2025. The children were randomly assigned to either an intervention (INT) or a control (CON) group by kindergarten classes. The present study employs a 2-group repeated-measures design to evaluate the effects of a 4-week (Experiment 1) and 10-week (Experiment 2) Wushu program on physical fitness and lower-limb strength in preschool children. The Wushu or game-based exercise programs were developed by a team of Wushu professors and exercise scientists in close collaboration with experienced kindergarten teachers. These programs were conducted with students from the Wushu or Athletic Training major. Prior to the commencement of the testing procedures, the participating children were acquainted with all aspects of the experimental protocol. The parents or guardians of the participants were made aware of the study procedures and provided written informed consent. The study protocol was approved by the Institutional Review Board of Shanghai University of Sport (102772023RT174, issued on 30 May 2023).

### 2.2. Participants

**Figure 1:**
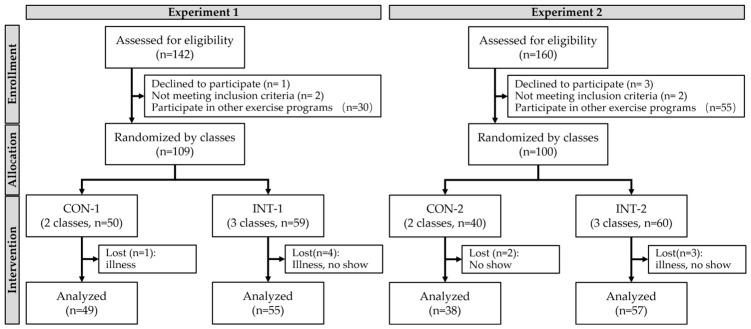
Participant flowchart.

A total of 199 children, aged between 5 and 6 years old, participated in this study. The children were enrolled in a kindergarten located in Shanghai, China, and randomly assigned to either an intervention (INT) or a control (CON) group, with the allocation made on a class-based basis. In 2024, Experiment 1 was conducted, in which 104 children completed the 4-week Wushu program (INT-1, 3 classes, n = 55) or a designed construction and carrying-based unstructured free play (CON-1, 2 classes, n = 49). In 2025, Experiment 2 was conducted, with 57 children (INT-2, 3 classes) participating in the 10-week Wushu exercise program. 38 children (CON-2, 2 classes) were in the control group. The sample size calculation was performed using R (version 4.3.2, R Foundation for Statistical Computing, Vienna, Austria). For a two-arm cluster randomized controlled trial, assuming an individual-level Cohen’s *d* of 0.8, an intraclass correlation coefficient (ICC) of 0.025, a two-sided α of 0.05, and 80% power. This yielded 15 participants per cluster. To account for up to 20% attrition, a minimum of 19 participants were recruited for each class. The inclusion criteria for participation in the study are as follows: (1) Age between 5 and 6 years; (2) good general health with no known history of neuromuscular, cardiovascular, or metabolic diseases; (3) no musculoskeletal injuries in the preceding 6 months; (4) ability to complete all physical fitness tests; and (5) provision of written informed consent from parents or legal guardians and verbal assent from the children. The exclusion criteria comprised the following: (1) The subject suffered from cardiovascular or respiratory diseases, or other unsuitable conditions for exercise; (2) participation in other structured exercise training programs during the study period; (3) unable to complete all physical fitness tests; and (4) unwillingness to participate by either the parent/guardian or the child. The participant flowchart t is presented in Figure 1.

### 2.3. Intervention

#### 2.3.1. INT Group

The Wushu exercise program administered to INT was delivered by professional Wushu athletes in collaboration with kindergarten teachers. Prior to the start of the intervention, a workshop and an informational session were made available to the participating parents and kindergarten teachers. The workshop and session covered the overall aim of the program, the content of the curriculum, key precautions, as well as the benefits and risk management for the exercise intervention. The Wushu exercise program (Zodiac Animal Kung Fu Boogie) incorporated twelve animal-themed movements derived from the Chinese zodiac, which were designed to enhance lower-limb muscular fitness. This program employed a playful and engaging approach to encourage participation. The movements primarily incorporate basic hand gestures, fundamental lower-body stances, and footwork patterns, along with trunk rotations, single-leg balances, and multidirectional jumps. A detailed description of the movements and the sets in the Wushu program is provided in Table 1. Each session lasted 30 min and consisted of warm-up, Wushu practice, and cool-down. The intervention was conducted over a period of 4 weeks in Experiment 1 and 10 weeks in Experiment 2, with three sessions per week.

#### 2.3.2. CON Group

The control group engaged in unstructured free play, with activities designed by exercise experts to ensure that lower-limb muscular fitness was targeted. The environment was equipped with a variety of materials, including building blocks, rollers, ladders, wooden boxes, and tires, allowing children to run, hop, and select from construction and carrying-based games. Supplementary Table S1 provided a summary of the activities and the targeted motor skills of the outdoor materials for the CON in kindergarten.

#### 2.3.3. Exercise Intensity Monitoring

The heart rate was monitored for children in both INT and CON groups during three randomly selected sessions, using an optical heart rate sensor (Polar Verity Sense, Kempele, Finland). The maximum heart rate (HRmax) was estimated using the following formula: 208 − 0.7 × age. The intensity of exercise was categorized as moderate when the heart rate was between 64% and 76% of HRmax, and as vigorous when it was between 77% and 95% of HRmax. An analysis of the monitoring data revealed that during the Wushu intervention session, the mean heart rate was 126 beats per minute (bpm), with a mean maximum heart rate of 158 bpm. The time spent at 60–69% HRmax and 70–79% HRmax accounted for 78% and 7% of the session, respectively, indicating that the exercise intensity of the Wushu program was predominantly moderate-to-vigorous. Monitoring data in CON showed that during the session, the mean heart rate was 135 bpm, with a mean maximum heart rate of 157 bpm. The proportion of time spent in the 60–69% HRmax and 70–79% HRmax zones was 84% and 6%, respectively, indicating that the exercise intensity for the control group was also predominantly moderate-to-vigorous and comparable to that of the Wushu intervention group.

### 2.4. Testing Procedures

Measurements were conducted at baseline and the day after the intervention period. Tests were carried out at the same time of the day by a group of trained students from Exercise and Health-related majors, who were blinded to group allocation to minimize measurement bias. Testing was performed within the kindergartens, with each child tested individually. The entire test procedure lasted approximately 30 min per child. Standardized verbal instructions and a visual demonstration were provided for each test, followed by a familiarization trial before the formal test trial.

#### 2.4.1. Primary Outcome

The primary outcome was lower-limb explosive power, which was assessed via SLJ [19,20]. The SLJ test was conducted on a firm, non-slip surface. The children were positioned behind the start line. Following preparatory countermovement and arm swing, they jumped forward by extending the hips, knees, and ankles, landing with both feet simultaneously while maintaining balance. The jump distance was measured from the start line to the nearest heel contact point. Each child performed two trials, and the maximum distance achieved was recorded for analysis.

#### 2.4.2. Secondary Outcomes

The coordination, speed, and agility, upper-limb strength, leg and back flexibility, and morphological indicators were assessed as the following secondary outcomes. The coordination was evaluated with the double-leg continuous jump test [19,20]. In this test, children performed 10 consecutive two-footed jumps over soft blocks placed in a line at 50 cm intervals. The start line is 20 cm before the first block, and the finish line is 20 cm after the last block. The speed and agility were measured using a 15 m zigzag run [20], where participants sprinted while weaving around seven cones, which were placed at 3, 4.5, 5, 7.5, 9, 10.5, and 12 m from the start line. The tests were manually timed on two attempts, and the faster time was recorded. The upper limb strength was examined through the handgrip strength test, with the highest value from two maximal-effort trials recorded in kilograms. The flexibility was assessed using the sit-and-reach test. Height and weight were also measured, and body mass index (BMI) was calculated. In Experiment 2, both SJ and CMJ were evaluated using a force platform (KWYP-FP6035-7K, Kunwei, Changzhou, China) with a sampling rate of 1000 Hz. For the SJ test, participants started from a static semi-squat position and jumped vertically without any preparatory countermovement. For the CMJ test, the subjects performed a rapid downward countermovement and then jumped with maximal effort. Two attempts were made for each jump test, and the maximum jump height was recorded. The EUR was calculated as the ratio of CMJ height to SJ height.

### 2.5. Statistical Analysis

Statistical analyses performed using R (version 4.3.2). Categorical variables of age and sex were summarized as frequencies and percentages, with between-group comparisons performed using Pearson’s Chi-squared test. The normality of continuous variables was assessed using the Shapiro–Wilk test. Data that follows a normal distribution is presented as the mean ± standard deviation (Mean ± SD), while data that does not follow a normal distribution is presented as the median (interquartile range). For normally distributed continuous variables with unequal variances between groups, the Welch Two-Sample *t*-test was applied. The Wilcoxon rank sum test (Mann–Whitney U test) was used for non-normally distributed continuous variables. A linear mixed-effects model (LMM) was used to analyze the data. The model included the baseline value, treatment group, age, and sex as fixed effects, and a random intercept for each cluster to account for within-cluster correlations. The model was fitted using restricted maximum likelihood. The statistical significance of the fixed effects was assessed through the implementation of *t*-tests, with the Kenward-Roger method for statistical adjustment. In addition, the Pearson correlation was used for exploring the relationships between these secondary outcomes at post-test in the INT-2 group and the primary outcomes of SLJ. A *p*-value < 0.05 was considered statistically significant.

## 3. Results

ICCs were estimated for all outcomes in two cluster randomized experiments (Supplementary Table S2). The majority were low, with 71.1% below 0.05 and 63.2% below 0.025. This indicates that individual-level variation typically dominates over cluster-level effects and that the bias from ignoring clustering would likely be minimal. However, ICCs were non-negligible for some measures. In Experiment 1, low ICCs ranging from 0.05 to 0.171 appeared for weight, grip strength, sit-and-reach, and standing long jump. Moderate ICCs, ranging from 0.232 to 0.239, were detected for BMI and the 15 m zigzag run. In Experiment 2, low ICCs ranging from 0.058 to 0.184 were observed for BMI, CMJ, double-leg continuous jump, sit-and-reach, standing long jump, and the zigzag run. Moderate ICCs ranging from 0.186 to 0.385 were identified for EUR, SJ, and grip strength. Therefore, LMMs were used for the clustered data structure to ensure a valid statistical analysis.

A total of 104 participants aged 5.82 ± 0.39 years maintained an attendance rate exceeding 85% in Experiment 1. The baseline characteristics of the participants are presented in Table 2. Prior to the intervention, no significant differences were observed in height (p=0.363), weight (p=0.155), or BMI (p=0.077) between CON-1 and INT-1. All pre-intervention physical fitness measures were also comparable, including grip strength (p=0.421), sit-and-reach (p=0.451), SLJ (p=0.602), double-leg continuous jump (p=0.079) and 15 m zigzag run (p=0.99). The estimated treatment effect is summarized in Table 3. After adjustment for baseline, class, age, and sex, the LMM revealed a statistically significant improvement of SLJ in INT-1 compared with CON-1. The marginal mean difference (estimate) was 5.95 (95% CI: 1.66 to 10.2), with a *p*-value of 0.007. No other significant differences were detected for height, weight, BMI, grip strength, sit-and-reach, double-leg continuous jump, 15 m zigzag run, or SJ height (all p>0.05).

In Experiment 2, ninety-five participants aged 5.42 ± 0.5 years demonstrated an attendance rate that exceeded 85%. The baseline characteristics are summarized in Table 4. The variables of height (p=0.231), weight (p=0.196), and BMI (p=0.69) did not differ between the CON-2 and INT-2. Similarly, the grip strength (p=0.495), sit and reach (p=0.429), SLJ (p=0.32), double-leg continuous jump (p=0.239), 15 m zigzag run (p=0.2), CMJ (p=0.351), SJ (p=0.262), and EUR (p=0.342) were all comparable. The estimated treatment effect is presented in Table 5. After adjusting for baseline values, class, age, and sex, the 10-week Wushu program significantly impacted explosive power, coordination, and agility metrics. Specifically, SLJ showed a marginal mean difference of 5.94 cm (95% CI: 0.103 to 11.8, *p* = 0.048). Double-leg continuous jump time significantly decreased by 1.04 s (95% CI: −1.45 to −0.634, *p* =0.005). The 15 m zigzag run time is improved by 0.645 s (95% CI: −1.25 to −0.04, *p* = 0.043), signifying enhanced agility and change-of-direction speed. In contrast, no significant group differences were observed for anthropometric measures (all p>0.05), grip strength (p=0.425), sit-and-reach (p=0.554), CMJ (p=0.224), SJ (p=0.728), or EUR (p=0.52).

**Figure 2:**
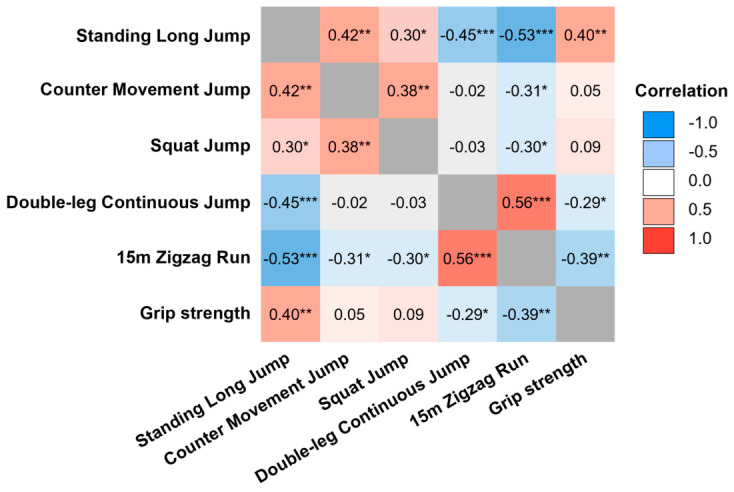
Pearson correlation analysis of muscular fitness−related indicators. The data was collected from the post−intervention INT−2 group. Colors in the heatmap represent the correlation strength: red for positive, blue for negative. *: *p* < 0.05; **: *p* < 0.01; ***: *p* < 0.001.

Pearson correlation analysis of post-intervention data from the INT-2 group revealed significant relationships between various physical performance measures (Figure 2). The study demonstrated a significant negative correlation between SLJ and 15 m zigzag run (*r* = −0.53, 95% CI: −0.7 to −0.31, *p* < 0.001), suggesting that an increase in SLJ distance is associated with enhanced agility performance. A moderate correlation was observed between SLJ and double-leg continuous jump (*r* = −0.45, 95% CI: −0.64 to −0.22, *p* < 0.001), CMJ height (*r* = 0.42, 95% CI: 0.18 to 0.61, *p* = 0.001), SJ height (*r* = 0.3, 95% CI: 0.05 to 0.52, *p* = 0.022), and grip strength (*r* = 0.4, 95% CI: 0.16 to 0.6, *p* = 0.002). Additionally, a robust positive correlation was identified between the duration of the double-leg continuous jump and 15 m zigzag run (*r* = 0.56, 95% CI: 0.35 to 0.71, *p* < 0.001). The 15 m zigzag run time exhibited a moderately negative correlation with grip strength (*r* = −0.39, 95% CI: −0.59 to −0.15, *p* = 0.003) and a weak negative correlation with CMJ height (*r* = −0.31, 95% CI: −0.53 to −0.06, *p* = 0.017) and SJ height *(r* = −0.3, 95% CI: −0.52 to −0.04, *p* = 0.024). A moderate positive correlation was observed between CMJ height and SJ height (*r* = 0.38, 95% CI: 0.13 to 0.58, *p* = 0.004). No significant correlations were observed between CMJ or SJ and double-leg continuous jump or grip strength (*p* > 0.05).

## 4. Discussion

The present study investigated the effects of Wushu exercise on the lower−limb explosive power and physical fitness in preschool children through two separate cluster−randomized trials of different durations. In both the 4−week and 10−week interventions, significant improvements were observed in SLJ performance. The 10−week intervention additionally enhanced double−leg continuous jump and 15 m zigzag run. The remaining indicators, including upper−limb strength and flexibility, along with the force plate−derived CMJ, SJ, and EUR, exhibited no significant change over the intervention period.

SLJ has been demonstrated to be a valid and sensitive index of lower−limb muscular fitness in youth, responding positively to training [5,6]. SLJ has gained widespread recognition as a practical and reliable field−based measure of lower−limb muscular strength and explosive power in this age group [21,22,23]. The findings from two independent Wushu intervention studies demonstrated that a 4−week and a separate 10−week Wushu intervention both significantly improved SLJ performance in preschool children. Furthermore, the 10−week intervention alone led to additional enhancements in performance on the double−leg continuous jump and the 15 m zigzag run. In the 10−week intervention, SJ, CMJ, and EUR demonstrated no alterations. The findings demonstrate that SLJ exhibited improvements following 4 and 10 weeks of Wushu training, thereby substantiating its responsiveness to alterations in muscular fitness. This observation is in alignment with the outcomes of prior exercise training studies conducted in young children [24].

The absence of an observed improvement in SJ suggests that Wushu exercises may not provide an adequate load to enhance maximal concentric strength in children. The absence of a change in both the CMJ and the EUR suggests that the intervention did not improve SSC efficiency to a level that could be discerned through these vertical jump tests. which could account for the observed improvement in SLJ performance. The lack of change in CMJ and EUR suggests that these measures were not responsive to the training stimulus in this group of children, which is consistent with previous reports of limited sensitivity in similar populations [25].

The improvements in double−leg continuous jump and zigzag run after 10 weeks, but not after 4 weeks, suggest that the dynamic actions involved in wushu, such as stopping, jumping, and landing, may have contributed to enhanced coordination. Wushu exercise is characterized by its incorporation of rapid directional changes, plyometric actions, and demands for interlimb coordination. The duration of programs enhancing agility and coordination is reported to be at least six to ten weeks in children [15,26]. In contrast, shorter interventions, with a duration of less than eight weeks, have been observed to be ineffective in improving change−of−direction ability, even among athletic youth populations [27,28]. The double−leg continuous jump further demonstrates the capacity for repeated lower−limb power generation and utilization of musculotendinous stiffness. Research has demonstrated the reliability and sensitivity of lower−limb stiffness measurements to medium− to long−term training [29]. Furthermore, combat sports have been shown to improve lateral continuous jump performance after several months [30]. These two tests are included in the Chinese National Physical Fitness Measurement for preschoolers [20] and reflect more complex motor skills that require coordination, rhythm, and rapid direction changes [31]. Therefore, the 10−week period was proven to be adequate for eliciting substantial enhancements in interlimb coordination and repeated force application.

The sit−and−reach flexibility of both groups remained unchanged, a finding that is consistent with a previous report on 5–6−year−old children following a 10−week Chinese martial arts intervention [3] and with a systematic review indicating that flexibility gains are not uniformly observed across martial arts studies [15].

In addition, we analyze the relationship between the SLJ and other strength− or lower−limb muscular fitness−related indicators. These correlations highlight distinct interrelationships between lower−body power, agility, and upper−body strength metrics post−intervention. SLJ, CMJ, and SJ are commonly used to assess lower−body muscular power and have established reliability and validity in children [5,32]. Grip strength is an indicator of overall muscular strength [33], while zigzag runs and continuous jumps assess agility and endurance components [34], as reported by others in adults and adolescents.

The present study has several limitations. First, randomization was performed at the class level rather than at the individual level. Despite the application of LMM with the Kenward−Roger method for small−sample cluster adjustments, the clustering effects have been shown to weaken observational independence and limit generalizability. Moreover, the two intervention durations were tested in separate cluster−randomized trials, without randomization between durations. Therefore, any cross−trial comparison is hypothesis−generating only and cannot establish a causal effect of duration. Future studies should therefore adopt individual−level randomization, consider designs with a larger number of clusters, such as multi−center trials, and incorporate repeated measures, including long−term follow−up to assess the sustainability of training effects. Secondly, the objective monitoring of physical activity occurring outside the designated research sessions was not recorded. Although we instructed children and parents not to practice the movements at home, the possibility that some children in the intervention group engaged in Wushu−related activities beyond the study cannot be excluded. Future work would benefit from using accelerometers to address this concern. Furthermore, the control activities were designed to provide comparable moderate−to−vigorous exertion, primarily involving lower−limb muscular fitness movements. However, they were not fully standardized or quantified. Consequently, it remains difficult to determine whether the observed benefits are attributable specifically to Wushu−related motor patterns or simply to differences between structured and unstructured physical activity exposure. Future research could compare Wushu with other structured activities that emphasize lower−limb muscular fitness, such as roller skating or small−sided soccer.

## 5. Conclusions

In conclusion, both the 4−week and 10−week Wushu programs were effective in enhancing lower−limb explosive power in children aged 5–6 years within their respective trials. The enhancements in coordination and jumping ability were only observed in the 10−week trial. These task−specific adaptations support the value of varied movement interventions for fostering comprehensive lower−limb motor competence in preschoolers.

## Figures and Tables

**Table 1:** Basic movements included in the lower-limb-muscular-fitness-dominated kindergarten Wushu program.

Basic Movements from ‘Zodiac Animal Kung Fu Boogie’ ^a^	Week 1	Week 2	Week 3	Week 4	Week 5	Week 6	Week 7	Week 8	Week 9	Week 10
Rat (Half Squat): Step diagonally forward and backward, squat quickly, and thrust punch.	3 × 3	3 × 1	3 × 2	3 × 2	3 × 2	3 × 3	3 × 1	3 × 2	3 × 2	3 × 2
Ox (Horse Stance): Step laterally left and right and thrust punch.	3 × 3	3 × 1	3 × 2	3 × 2	3 × 2	3 × 3	3 × 1	3 × 2	3 × 2	3 × 2
Tiger (High Lunge): Step laterally left and right, pivot, and form tiger claws.	3 × 3	3 × 1	3 × 2	3 × 2	3 × 2	3 × 3	3 × 1	3 × 2	3 × 2	3 × 2
Rabbit (Vertical Jump): Hop on one leg, left and right, then form rabbit fists.	3 × 3	3 × 1	3 × 2	3 × 2	3 × 2	3 × 3	3 × 1	3 × 2	3 × 2	3 × 2
Dragon (Lunge): Step laterally left and right, squat, and form dragon claws.	3 × 2	3 × 3	3 × 1	3 × 2	3 × 2	3 × 2	3 × 3	3 × 1	3 × 2	3 × 2
Snake (Kneeling Stance): Step laterally left and right, perform a single-leg kneeling squat, and form snake hands.	3 × 2	3 × 3	3 × 1	3 × 2	3 × 2	3 × 2	3 × 3	3 × 1	3 × 2	3 × 2
Horse (Half Squat): Perform a single-leg standing squat, imitating a horse-riding posture.	3 × 2	3 × 3	3 × 1	3 × 2	3 × 2	3 × 2	3 × 3	3 × 1	3 × 2	3 × 2
Goat (Rotational Jump): Perform lateral rotational jumps left and right, assume a high horse stance, and pivot.	3 × 2	3 × 3	3 × 1	3 × 2	3 × 2	3 × 2	3 × 3	3 × 1	3 × 2	3 × 2
Monkey (Step Forward): Step forward, perform a back kick, and execute monkey fist.	3 × 1	3 × 2	3 × 3	3 × 2	3 × 2	3 × 1	3 × 2	3 × 3	3 × 2	3 × 2
Rooster (Single-Leg Stance): Stand on one leg, extend arms sideways, imitating a golden rooster.	3 × 1	3 × 2	3 × 3	3 × 2	3 × 2	3 × 1	3 × 2	3 × 3	3 × 2	3 × 2
Dog (Dragging Step): Perform a single-leg kneeling squat, pivot, and execute a back kick.	3 × 1	3 × 2	3 × 3	3 × 2	3 × 2	3 × 1	3 × 2	3 × 3	3 × 2	3 × 2
Pig (High Horse Stance): Hold an imaginary Tai Chi ball.	3 × 1	3 × 2	3 × 3	3 × 2	3 × 2	3 × 1	3 × 2	3 × 3	3 × 2	3 × 2

^a^: The program was conducted for 4 weeks in experiment 1 (week 1 to week 4) and 10 weeks in experiment 2 (week 1 to week 10). Basic Movements were expressed as sessions × sets per week.

**Table 2:** Baseline characteristics in Experiment 1.

Variable	Overall ^a^ (N = 104)	CON-1 ^a^ (N = 49)	INT-1 ^a^ (N = 54)	*p*-Value
Age				0.981 ^b^
5 (yrs)	19 (18.3%)	9 (18.4%)	10 (18.2%)	
6 (yrs)	85 (81.7%)	40 (81.6%)	45 (81.8%)	
Sex				0.686 ^b^
female	51 (49.0%)	23 (46.9%)	28 (50.9%)	
male	53 (51.0%)	26 (53.1%)	27 (49.1%)	
Height (cm)	119.2 ± 4.6	119.6 ± 4.5	118.8 ± 4.8	0.363 ^c^
Weight (kg)	22.2 (20.3, 25.2)	22.9 (21.0, 26.0)	21.2 (20.1, 24.7)	0.155 ^d^
BMI (kg/m^2^)	15.71 (14.99, 16.89)	15.96 (15.36, 17.16)	15.50 (14.82, 16.66)	0.077 ^d^
Grip strength (kg)	10.50 (9.50, 12.00)	10.50 (9.50, 11.50)	11.00 (9.50, 12.00)	0.421 ^d^
Sit-and-Reach (cm)	9.2 ± 5.5	8.7 ± 5.7	9.5 ± 5.4	0.451 ^c^
Standing Long Jump (cm)	102.07 ± 15.2	101.22 ± 17.22	102.82 ± 13.27	0.602 ^c^
Double-leg Continuous Jump (s)	5.70 (5.16, 6.40)	6.03 (5.19, 6.68)	5.50 (5.16, 6.15)	0.079 ^d^
15 m Zigzag Run (s)	6.84 (6.50, 7.37)	6.81 (6.53, 7.49)	6.91 (6.38, 7.31)	0.99 ^d^

^a^: n (%); Mean ± SD; Median (Q1, Q3); ^b^: Pearson’s Chi-squared test; ^c^: Welch Two Sample *t*-test; ^d^: Wilcoxon rank sum test. Yrs: years. cm: centimeters. m: meters. kg: kilograms. s: seconds.

**Table 3:** Estimated effect of a 4-week Wushu Program (Experiment 1).

Variable	Change from Baseline ^a^		Marginal Mean of Change ^b,c^		Marginal Mean Difference (95% CI) ^c^	*t*−Value	*p*−Value
	CON−1 (N = 49)	CON−1 (N = 49)	CON−1 (N = 49)	INT−1 (N = 55)			
Height (cm)	0.8 ± 1.68	0.8 ± 2.32	0.74 ± 0.35	0.66 ± 0.32	−0.06 (−0.87, 0.75)	−0.152	0.879
Weight (kg)	0 ± 0.77	−0.2 ± 1.56	−0.19 ± 0.21	−0.47 ± 0.20	−0.27 (−0.75, 0.21)	−1.129	0.262
BMI (kg/m^2^)	−0.2 ± 0.7	−0.3 ± 0.83	−0.33 ± 0.13	−0.48 ± 0.12	−0.16 (−0.45, 0.14)	−1.039	0.302
Grip strength (kg)	0.37 ± 2.16	0.38 ± 2.14	0.15 ± 0.31	0.38 ± 0.29	0.23 (−0.50, 0.95)	0.619	0.537
Sit−and−Reach (cm)	0.84 ± 4.44	0.21 ± 3.23	0.35 ± 0.62	−0.10 ± 0.58	−0.45 (−1.89, 1.00)	−0.615	0.540
Standing Long Jump (cm)	1.33 ± 12.97	6.4 ± 12.12	0.75 ± 1.86	6.70 ± 1.71	5.95 (1.66, 10.24)	2.754	0.007
Double−leg Continuous Jump (s)	−0.72 ± 1.1	−0.57 ± 0.7	−0.51 ± 0.12	−0.58 ± 0.11	−0.07 (−0.34, 0.20)	−0.497	0.620
15 m Zigzag Run (s)	−0.57 ± 0.66	−0.64 ± 0.75	−0.50 ± 0.10	−0.57 ± 0.10	−0.07 (−0.26, 0.13)	−0.678	0.499

^a^: Mean ± SD; ^b^: Mean ± SE; ^c^: Based on a linear mixed-effects model (LMM) after adjusting for baseline, age, and sex; random effects: Class. CI: Confidence Interval. SD: Standard Deviation. SE: Standard Error.

**Table 4:** Baseline characteristics in Experiment 2.

Variable	Overall ^a^ (N = 95)	CON-2 ^a^ (N = 38)	INT-2 ^a^ (N = 57)	*p*-Value
Age				0.913 ^b^
5 (yrs)	78 (82.1%)	31 (81.6%)	47 (82.5%)	
6 (yrs)	17 (17.9%)	7 (18.4%)	10 (17.5%)	
Sex				0.104 ^b^
female	38 (40.0%)	19 (50.0%)	19 (33.3%)	
male	57 (60.0%)	19 (50.0%)	38 (66.7%)	
Height (cm)	116.3 ± 4.3	115.6 ± 4.3	116.7 ± 4.3	0.231 ^c^
Weight (kg)	21.40 (19.00, 23.20)	20.80 (18.70, 22.80)	21.80 (19.00, 24.00)	0.196 ^d^
BMI (kg/m^2^)	15.48 (14.49, 16.82)	15.41 (14.57, 16.35)	15.58 (14.49, 16.86)	0.69 ^d^
Grip strength (kg)	9.00 (7.00, 10.50)	8.75 (6.00, 10.50)	9.00 (7.00, 10.00)	0.495 ^d^
Sit-and-Reach (cm)	9.0 ± 5.1	9.5 ± 5.8	8.6 ± 4.5	0.429 ^c^
Standing Long Jump (cm)	95 ± 18.21	92.61 ± 20.56	96.6 ± 16.45	0.32 ^c^
Double-leg Continuous Jump (s)	5.91 (5.25, 7.32)	5.74 (4.94, 7.38)	6.09 (5.56, 7.13)	0.239 ^d^
15 m Zigzag Run (s)	7.00 (6.59, 7.60)	7.21 (6.62, 7.87)	6.94 (6.56, 7.29)	0.2 ^d^
CMJ (cm)	14.2 ± 4.4	13.7 ± 4.2	14.5 ± 4.5	0.351 ^c^
SJ (cm)	9.2 (7.0, 11.5)	9.2 (6.7, 10.9)	9.3 (7.5, 11.6)	0.262 ^d^
EUR	0.64 (0.51, 0.82)	0.64 (0.49, 0.78)	0.64 (0.52, 0.83)	0.342 ^d^

^a^: n (%); Median (Q1, Q3); ^b^: Pearson’s Chi-squared test; ^c^: Welch Two Sample *t*-test; ^d^: Wilcoxon rank sum test. Yrs: years. cm: centimeters. m: meters. kg: kilograms. s: seconds.

**Table 5:** Estimated effect of a 10−week Wushu Program (Experiment 2).

Variable	Change from Baseline ^a^		Marginal Mean of Change ^b,c^		Marginal Mean Difference (95% CI) ^c^	*t*−Value	*p*−Value
	CON−2 (N = 38)	INT−2 (N = 57)	CON−2 (N = 38)	INT−2 (N = 57)			
Height (cm)	1.02 ± 0.51	1.16 ± 0.68	1.04 ± 0.25	1.09 ± 0.21	0.05 (−0.94, 1.04)	0.162	0.882
Weight (kg)	1.30 ± 0.81	1.31 ± 0.74	1.35 ± 0.20	1.34 ± 0.17	−0.01 (−0.79, 0.78)	−0.038	0.972
BMI (kg/m^2^)	0.69 ± 0.64	0.63 ± 0.5	0.69 ± 0.10	0.65 ± 0.09	−0.04 (−0.44, 0.36)	−0.348	0.753
Grip strength (kg)	0.53 ± 1.96	−0.11 ± 1.73	0.42 ± 0.59	−0.27 ± 0.50	−0.69 (−3.11, 1.72)	−0.924	0.425
Sit−and−Reach (cm)	1.14 ± 5.06	1.32 ± 4.01	1.35 ± 0.57	0.90 ± 0.51	−0.45 (−2.64, 1.74)	−0.668	0.554
Standing Long Jump (cm)	1.87 ± 7.09	7.25 ± 6.81	1.10 ± 1.48	7.04 ± 1.27	5.94 (0.10, 11.78)	3.243	0.048
Double−leg Continuous Jump (s)	−0.09 ± 1.01	−1.33 ± 1.14	−0.12 ± 0.10	−1.16 ± 0.09	−1.04 (−1.45, −0.63)	−8.537	0.005
15 m Zigzag Run (s)	−0.29 ± 0.5	−0.91 ± 0.48	−0.24 ± 0.15	−0.89 ± 0.13	−0.64 (−1.25, −0.04)	−3.388	0.043
CMJ (cm)	4.45 ± 4.00	5.77 ± 4.55	3.80 ± 1.08	5.88 ± 0.90	2.08 (−2.26, 6.43)	1.528	0.224
SJ (cm)	2.54 ± 5.66	1.11 ± 6.07	2.55 ± 2.24	1.46 ± 1.86	−1.09 (−10.18, 8.00)	−0.382	0.728
EUR	−0.02 ± 0.42	−0.17 ± 0.39	−0.01 ± 0.13	−0.13 ± 0.11	−0.12 (−0.64, 0.40)	−0.726	0.520

^a^: Mean ± SD; ^b^: Mean ± SE; ^c^: Based on a linear mixed−effects model (LMM) after adjusting for baseline, age, and sex; random effects: Class. CI: Confidence Interval. SD: Standard Deviation. SE: Standard Error.

## Data Availability

The data is available on request from the corresponding author. The data contains personal information of child participants, and the IRB has restricted public data sharing to protect their privacy.

## Supplementary Materials

The following supporting information can be downloaded at: https://www.mdpi.com/article/10.3390/jfmk11020222/s1. Table S1. Outdoor free play materials in kindergarten; Table S2. Intraclass correlation coefficients (ICCs) for the assessed variables.

## Abbreviations

The following abbreviations are used in this manuscript:

BMI	Body Mass Index
CMJ	Countermovement Jump
CON	Control
EUR	Eccentric Utilization Ratio
HRmax	Maximum Heart Rate
INT	Intervention
SJ	Squat Jump
SLJ	Standing Long Jump
