# Supplementary material for: Effects of Wushu Programs on Lower-Limb Explosive Power in Preschool Children Aged 5–6 Years: A Cluster-Randomized Controlled Trial
Source: J Funct Morphol Kinesiol. 2026 May 31;11(2):222. doi: 10.3390/jfmk11020222 (PMC13301314; doi:10.3390/jfmk11020222)
Supplement: Supplementary file 1 [file jfmk-11-00222-s001.zip › jfmk-4326166-supplementary.pdf]

Supplement Table S1. Outdoor free play materials in kindergarten

Material	Lower-limb muscular fitness activities	Motor Skills
Wooden boxes	Children were observed jumping onto and off crates, stacking crates and climbing over them, and carrying crates while walking or running between play areas.	Lower-limb explosive power, coordination, spatial orientation
Wooden platform	Children spontaneously climbed onto high platforms, jumped down from various heights, and stepped on/off repeatedly.	Lower-limb strength, jumping ability
Long planks	In free play, children used planks as ramps, balance beams, or low swing frames pushing the ground with legs.	Balance, core stability, lower body strength
Rollers	Children were seen standing or walking on rolling barrels, often with peer support, pushing/pulling heavy barrels, and crawling through them. Hopping over barrels was also naturally attempted.	Dynamic balance, limb coordination, core strength
Mats	Mats were used as cushioning for jumping from heights, rolling, and landing. Children performed squat jumps, tuck jumps, and practiced landing softly.	Flexibility, self-protection, body control
Tires	Children naturally carried tires, stacked them into makeshift steps, created obstacle courses for hopping in and out of tires, and ran around tire piles.	Limb coordination, endurance
Scooter boards	Children sat or knelt on scooter boards and propelled themselves with legs pushing off the ground down gentle slopes or on flat surfaces.	Dynamic balance, core stability, reaction speed
Ladders	Children climbed ladders placed horizontally or leaning against structures. They also combined ladders with boxes to create jumping challenges.	Upper-limb strength, hand-foot coordination, spatial orientation

Material	Lower-limb muscular fitness activities	Motor Skills
Ropes	Children were observed using ropes for assisted climbing or in group games requiring stable leg stances.	Grip strength, arm strength, teamwork
Large building blocks	Children carried, stacked, and built structures with blocks. They squatted repeatedly, lifted with legs, and walked while balancing blocks.	Upper-limb and shoulder strength, hand-eye coordination
Large pipes	Children carried large pipes together, or walking alongside. They also stepped over pipes or balanced them while running.	Gross muscle strength, endurance, teamwork

Supplement Table S2. Intraclass correlation coefficients (ICCs) for the assessed variables

Variable	Time	Experiment 1		Experiment 2	
		CON-1 (N=49)	INT-1 (N=55)	CON-2 (N=38)	INT-2 (N=57)
**Height (cm)**	Pre	0.010	0.000	0.000	0.000
**Height (cm)**	Post	0.000	0.000	0.000	0.000
**Weight (kg)**	Pre	0.155	0.000	0.000	0.000
**Weight (kg)**	Post	0.171	0.002	0.000	0.000
**BMI (kg/m^2)**	Pre	0.081	0.000	0.047	0.047
**BMI (kg/m^2)**	Post	0.232	0.000	0.086	0.019
**Grip strength (kg)**	Pre	0.147	0.000	0.000	0.249
**Grip strength (kg)**	Post	0.000	0.000	0.035	0.000
**Sit-and-Reach (cm)**	Pre	0.000	0.088	0.000	0.000
**Sit-and-Reach (cm)**	Post	0.105	0.011	0.094	0.000
**Standing Long Jump (cm)**	Pre	0.015	0.000	0.000	0.000
**Standing Long Jump (cm)**	Post	0.056	0.000	0.058	0.010
**Double-leg Continuous Jump (s)**	Pre	0.020	0.040	0.080	0.046
**Double-leg Continuous Jump (s)**	Post	0.014	0.000	0.000	0.000
**15m Zigzag Run (s)**	Pre	0.000	0.239	0.000	0.005
**15m Zigzag Run (s)**	Post	0.000	0.114	0.000	0.184
**CMJ (cm)**	Pre	N/A	N/A	0.000	0.013
**CMJ (cm)**	Post	N/A	N/A	0.000	0.172
**SJ (cm)**	Pre	N/A	N/A	0.000	0.130
**SJ (cm)**	Post	N/A	N/A	0.369	0.195
**EUR**	Pre	N/A	N/A	0.032	0.012
**EUR**	Post	N/A	N/A	0.385	0.186
